# Self-Healing Composites: A Path to Redefining Material Resilience—A Comprehensive Recent Review

**DOI:** 10.3390/ma17194681

**Published:** 2024-09-24

**Authors:** Maria Luísa Durão, Luís Nobre, Carlos Mota, João Bessa, Fernando Cunha, Raúl Fangueiro

**Affiliations:** 1 Fibrenamics, University of Minho, 4800-058 Guimarães, Portugal; 2 Department of Textile Engineering, University of Minho, Campus de Azurem, 4800-058 Guimarães, Portugal

**Keywords:** polymer matrix composites, smart materials, material resilience, self-healing

## Abstract

Polymeric composites are prone to undergoing damage, such as microcracks, during their operation, which can ultimately lead to catastrophic failure. To contradict such a problem, efforts have been carried out, by the scientific community, towards developing self-healing composites that, by mimicking biological systems, can autonomously and prematurely repair flaws, extending the durability and improving the security of materials. The present review explores the progress made in this area, focusing on extrinsic self-healing methods, as these can be employed to a variety of materials. Reservoir-based techniques, which resort to capsules, hollow fibers or microvascular networks, and thermoplastic-based ones are overviewed, prioritizing innovative approaches made in recent years. At last, promising practical applications for self-healing composites are highlighted and future challenges and opportunities are pointed out.

## 1. Introduction

**Figure 1:**
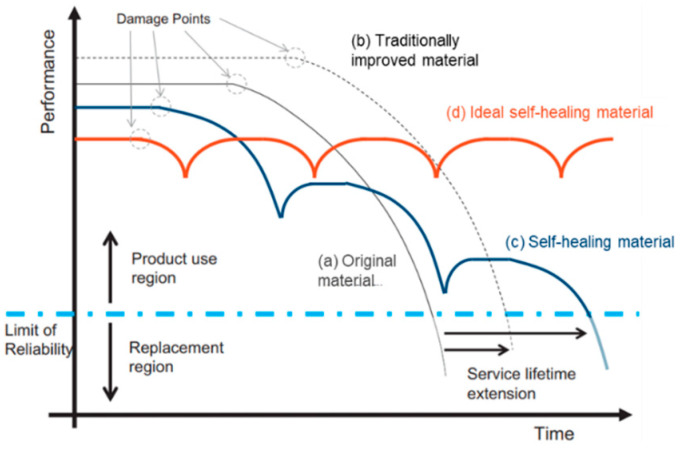
Comparison between the lifetime expansion of self-healing materials and traditional ones. Adapted from “Effect of polymer architecture on the intrinsic self-healing character of polymers” by Santiago J. Garcia, used under CC BY 4.0 [https://doi.org/10.1016/j.eurpolymj.2014.01.026] [17].

Self-healing polymer matrix composites, as the name suggests, are materials able to, when subjected to external damage, autonomously repair the affected area without manual intervention, and recover their integrity and functional properties (for example, strength, conductivity or biomedical characteristics), thus mimicking biological regeneration systems. In addition to the self-healing characteristics, these materials can still incorporate fillers responsible for providing other properties, like reinforcing fibers. The aforementioned damages include not only small imperfections caused, for example, by micro delamination of the incorporated fibers or by microcracking, but also fractures or other damage resulting from wear and environmental factors, such as pressure, impact or mechanical and thermal loads, and other defects derived from photodegradation, corrosion or chemical abrasion. These defects formed either during fabrication or during service life, if not detected and repaired in time, can compromise the integrity of the material, potentially leading to more severe damage and, ultimately, catastrophic failure. Most structural and high-performance composites are constituted by a thermosetting matrix that entails challenges when it comes to repair; for that reason, self-healing becomes relevant, particularly in the case of microfractures that occur within the structure of materials and are therefore difficult to detect and repair. Hence, with this approach, it is possible to assess damage at an early stage and therefore increase the service life of composites, as the graph in Figure 1 schematizes, reduce the need for maintenance, as well as the associated costs, improve the safety of structures and increase the possibilities of less conservative designs. In light of this, an ideal self-healing composite (Figure 1) should be able to naturally, repeatedly and consistently fix itself to the extent of considerable, but not too large, damage, regardless of its size [1,2,3,4,5,6,7,8,9,10,11,12,13,14,15,16,17].

The relevance of self-healing capacity is therefore clear, considering the demand for functional materials for modern applications. Thus, the market of self-healing materials in general (not specifically self-healing polymer matrix composites) is expected to grow in a compound annual growth rate of 56.5% from 2021 to 2029. Within this context, the construction segment will be the largest consumer of these materials, holding approximately 55.5% of the market, and it is expected to undergo the fastest growth during the analyzed period. Despite that, automotive and electronic device manufacturing industries are expected to endorse growth altogether with paints and coatings [18].

It is against this background of growing research interest in self-healing composites that this paper was written, aiming to explore and summarize the most recent and innovative developments on the field and to highlight how such materials can redefine the future path of material resilience by offering substantial advantages over traditional repair methods, including extended lifespan, reduced maintenance costs, safety, reliability and damage tolerance, as well as a significant contribution to sustainability. A review of the different approaches to self-healing is provided, focusing on the working principles and their main strengths and weaknesses, and supported by relevant experimental works elaborated in recent years. Potential applications of the studied materials are also highlighted, together with the challenges and opportunities of this field.

## 2. Approaches to Self-Healing

**Figure 2:**
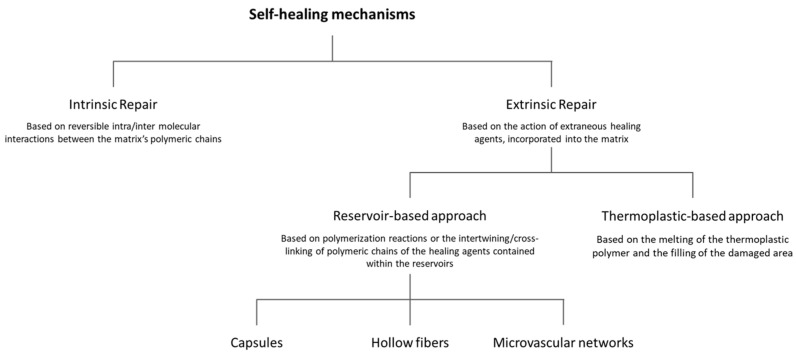
Synthesis of the mechanisms of self-healing.

The mechanism of self-healing (Figure 2) can be divided into two major types: intrinsic repair, which occurs through reversible intra- and/or intermolecular interactions between the polymeric chains of the material itself (such as dynamic non-covalent bonds or hydrogen bonding), and extrinsic repair, which occurs through the action of extraneous healing agents that are, in turn, incorporated into the composite’s matrix, which does not have the repairing capacity itself. For this last case, the response is either based on polymerization reactions, catalyzed or not, such as ring-opening or cationic polymerization; or on the intertwining or cross-linking of the polymer chains by the action of curing agents. Another extrinsic repair response includes the incorporation of thermoplastic polymers [1,4,14,15,19,20]. Extrinsic self-healing stands out for its high healing efficiency, low cost and possibility to be implemented in a more diverse range of materials, whilst intrinsic self-healing is limited to materials with inherent reversible molecular interactions that need rigorous reaction conditions and are therefore tendentially dependent on external stimulus (such as temperature or UV light) to act. In addition, it is also limited to smaller volumes of damage, as the repair occurs on a molecular scale. Nevertheless, intrinsic self-healing also possesses advantages, such as repeatability of self-repair, easiness of application and fewer limitations concerning integration and compatibilization of the healing agents [6,11,12,15,21,22,23,24,25,26]. This review primarily focuses on extrinsic self-healing due to its versatility and the potential to extrapolate its principles to a wider range of materials through the tailoring of the healing agents to specific needs, making scale-up and integration into existing technologies more viable. Moreover, this approach also offers the advantage of allowing the system to act independently [11,14,16].

Although the response of the repair process itself is, in part, similar for the different approaches to extrinsic self-healing, the mechanism will rely on the chemical groups present around the fracture, which, in turn, differ whether it involves only the matrix material or both the matrix and other fillers. Thus, the effectiveness of the process depends on the reactivity of the healing agent with the surfaces it comes in contact with. The material’s relaxation time is also considered crucial to the repair mechanism, assuming that slow relaxation is favorable to the self-repair mechanism, as it contributes to the rearrangement of polymer chains [4].

### 2.1. Healing Efficiency

When developing a self-healing composite, its performance should be evaluated and quantified. Therefore, repair efficiency is preferably calculated, which often consists of the ratio between the mechanical properties of the material before and after repair or of the self-healed material compared to the virgin one (Equation (1)). Nevertheless, some authors may also consider other alternative formulas where the property of the material after it has been damaged but before healing is subtracted from both the numerator and the denominator. Ergo, a perfectly healed material would have an efficiency of 1 (*η* = 1). This variable can be defined based on several mechanical properties, some examples being fracture resistance, fracture stress, material strength or moduli related to mechanical properties (e.g., Young’s modulus) [2,4,27,28]. An adequate property ought to be chosen according to the final purpose of the composite; for example, impact strength is particularly important when it comes to fiber composites for aerospace and military sectors [22,28]. The authors believe that the first option is preferable, as incorporating materials responsible for self-healing performance into the base material is likely to modify the materials’ virgin properties. Therefore, it would be more appropriate to use the property of the original self-healing material, rather than the property of the virgin one, when evaluating how much the material can self-heal. Even so, it remains important to compare these two properties, in other words, the properties before and after the conferment of the self-healing characteristics [28].

Nevertheless, the fact that the self-healing composites’ efficiency is not determined in a standard way in all studies, especially regarding the mechanical property under analysis and the extent of the damage, makes the comparison between the different methods under development non-linear [28].

(1) $$\eta =\frac{Property_{self\text{-}healed\;material}}{Property_{original\;material}}\;or\;\;\eta =\frac{Property_{self\text{-}healed\;material}}{Property_{virgin\;material}}$$

### 2.2. Self-Healing through Reservoirs

**Figure 3:**
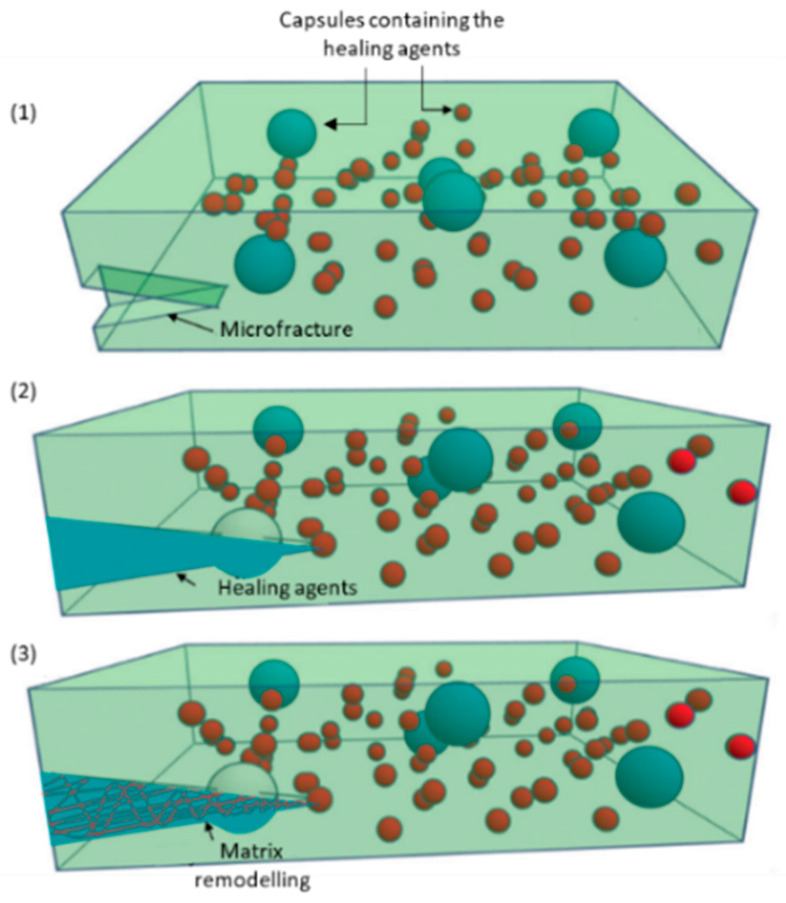
Healing process using repair agents stored in reservoirs; in this example, capsules are used (Blue and red spheres): (**1**) fracture formation; (**2**) release of repair agents contained in the reservoirs; (**3**) reparation of the matrix. Adapted from “Progress and challenges in self-healing composite materials” by Shafiqul Islam and Gajanan Bhat, used under CC BY 3.0. [https://doi.org/10.1039/D0MA00873G] [1].

The reparative response through reservoirs incorporated into the matrix occurs in three main steps, as shown in Figure 3: first, the driving action, that is, the damage that will lead to the breakdown of the reservoirs and consequent release of the repair compounds contained therein, followed by their transport to the site of damage, for example, the crack, and finally the chemical repair of the matrix with the aim of restoring the structural integrity of the material [1,12,19,25].

There are diverse types of structures for these reservoirs, particularly capsules, hollow fibers and capillary networks, typically consisting of an inert material compatible with the matrix and intended to protect the repairing agents from unwanted reactions or changes on their chemical properties. Generally, to achieve an efficient self-healing composite, it is necessary to consider some important factors concerning the reservoirs, namely, strength, which should ensure safe handling and processing and good storage stability, so that no deformation takes place and breakage only happens when the material’s damage occurs, and excellent interface adhesion, which guarantees that when the fracture is formed, the container ruptures and the healing agent is released. In some cases, compatibilizers might be necessary for that purpose. Likewise, the incorporation of the reservoirs in the matrix is relevant, considering that they must be properly embedded and uniformly distributed [1,2,21,29,30]. Ultimately, the quantity, shape and size of these reservoirs can be adjusted to the material’s final application [27].

When it comes to the healing agents, they should ensure not only high reactivity, but also good flowability and adhesion/compatibility with the polymeric matrix, despite the possibility of either being different from the matrix or not [29]. Additionally, such reactivity must allow them to also react quickly and effectively regenerate the composite material in question. Other relevant properties of these reactants include good storage stability within the structures that contain them so that they do not react undesirably, degrade at common composites’ manufacturing temperatures or entail safety risks. Furthermore, surface tension, contact angle and permeability must be adequate so that the diffusion process is efficient and gaps are quickly filled [1,4,31].

In the early development stages of extrinsic self-healing composites, one pioneer approach was the employment of dicyclopentadiene, which undergoes ring-opening metathesis polymerization catalyzed by Grubbs catalyst. However, in recent years, research has been dedicated to developing more efficient healing agents, not only based on catalyzed ring-opening polymerization, such as 5-ethylene-2-norbornene, which exhibits a superior healing efficiency, but also resorting to polymers similar to the matrix, as is the case of epoxy resins combined with curing agents [8,29]. These stand out due to their better affinity with the mentioned matrix and lower curing temperature, resulting in superior self-healing potential. However, they are also more viscous and less reactive than catalyst-based systems and can, therefore, create limitations concerning processability [29].

To promote a better performance of the reservoir-based self-healing systems, the possibility of using solvents has also been addressed either to facilitate the transport of the catalyst [32] or to penetrate the polymeric matrix, weaken the intermolecular forces, intensify the movement of the polymeric chains and lead to swelling of the material, which will improve contact between the fracture components and facilitate repair [33]. Furthermore, for systems where encapsulated resins are used, it should be considered that, even if the curing agent can react at room temperature, the viscosity of the resin, under these conditions, can be a limitation when it comes to its ability to infiltrate fractures.

Other nanomaterials, such as carbon nanotubes, can also be stored inside the reservoirs as an additional component that can not only promote the reinforcement of the newly solidified polymer (either in a resin/hardener system or on a monomer/initiator one), improving the mechanical properties’ recovery, but also potentially contribute to the restoration of other functional properties, such as electrical conductivity [34,35]. This is exemplified by the work developed by Hasna Hena Zamal et al. [13], where a mixture of carbon nanotubes with the healing agent stored inside the reservoirs restored 82% of the electrical conductivity.

#### 2.2.1. Capsules

Capsule-based self-healing composites (Figure 3) (Table 1) are the most extensively studied ones, particularly the ones that resort to microcapsules, due to the variety and the ease of implementation in different polymeric systems, resulting in a thorough description of the fabrication methods. Capsules can either be single, with, for example, the catalyst embedded in the matrix, or double, containing more than one regenerating reactant in the same capsule or in different capsules [1].

Within this context, H. Hu et al. [36] explored the development of an epoxy-resin-based composite, where two different types of microcapsules were used: one containing the same polymer as the matrix (12 wt%) and the other containing an amine curing agent (8 wt%) that would react with the first one. The healing conditions consisted of 24 h at room temperature, followed by 6 h, at 50 °C. It was concluded that the healing efficiency, evaluated through the fracture toughness, was about 80.4%. Alternatively, C. Y. Zhang et al. [37] proposed the encapsulation of styrene and benzoyl peroxide separately, so that the first was polymerized through initiation with the second upon microcapsule breakage. Such reservoirs were incorporated into an epoxy-based matrix and a maximum healing efficiency of 65% was attained, through impact tests, when 15 wt% of styrene loaded capsules and 3 wt% of benzoyl peroxide loaded capsules were employed and after 24 h, at 25 °C. The materials’ multi-repairability was also analyzed and a reduction of about 20% to 30% was detected for the second and third repairs. On a different approach, H. H. Zamal et al. [38] encapsulated a mixture of monomers of 5-ethylidene-2-norbornene with multi-walled carbon nanotubes, which were then dispersed (10 wt%) in an epoxy polymeric matrix, together with a ruthenium Grubbs catalyst, responsible for initiating ring opening metathesis polymerization reaction. It was highlighted that an ambient temperature curing resin was chosen for the matrix to prevent catalyst degradation. The main conclusions were, firstly, that the presence of the microcapsules contributed to the reinforcement of the material, and, secondly, that the carbon nanotubes increased the strength of the polymer synthesized upon capsule rupture, which was reflected on an improved healing efficiency of 97%, calculated by fracture toughness, after healing at room temperature for 48 h. Another distinct concept worth analyzing is the one developed by R. Rodriguez et al. [39], where microcapsules (20 wt%) containing an epoxy resin (the same as the matrix) on the inside and a curing initiator catalyst (Scandium triflate (III)) on the outer surface were prepared. This type of structure amplified the probability of the encapsulated resin reacting with the catalyst and, consequently, an efficiency of 79.1% was attained after healing at 120 °C, for 24 h, analyzed in terms of fracture toughness recovery.

In the particular case of sandwich composites, which are widely used in many applications, of which wind-turbines are an example, transverse load-induced impact damages can occur, critically affecting the sandwich core, often made of polymer foam. Such damages may compromise the overall performance of the composite; therefore, incorporating self-healing microcapsules within the polymer core or even in the interface layers can be beneficial for maintaining the integrity of these materials [40]. With that in mind, Shunze Cao et al. [23] explored the utilization of multicore-like bilayer calcium-alginate capsules, containing epoxy resin on the inner core, while a curing agent was on the outer one. Such structures were synthesized through multi-stage encapsulation and incorporated (15% volume fraction) into epoxy-based foams whose purpose was to integrate foam core sandwich structures. The advantage of this type of microcapsules was their capability to promote a more adequate mixture of the two healing agents. It was suggested that, when compared to conventional dual capsule systems, the multicore capsules exhibited better multiple self-healing performance, with a maximum efficiency greater than 80% for the first cycle, determined through the elastic modulus. Nevertheless, this efficiency decreased throughout the cycles. Moreover, the compressive strength and stiffness of the foams were improved.

On their work, Amanda R. Jones et al. [41] explored the self-healing of carbon fiber/epoxy interfaces, aiming to prevent fiber/matrix debonding, which has consequences for the mechanical properties of the material. For that purpose, micro-capsules containing a resin solvent solution responsible for interface regeneration were incorporated into the reinforcing carbon fibers’ surface. Upon debonding, the microcapsules underwent rupture, releasing the healing agents that repaired the interface. The usage of a binder to stabilize the capsules on the fibers’ surface was assessed, as well as the impact of the coverage of microcapsules at the interface. It was concluded that a maximum of 91% recovery of the interfacial shear strength could be achieved after 24 h at room temperature. Finally, an innovative concept was introduced by Wrihao Yuan et al. [42] that, aside from incorporating microcapsules containing epoxy resin and mercaptan into the polymeric matrix, modified silver nanoparticles on the surface of the reinforcing carbon fibers. The purpose of such an approach was that the reaction between the epoxy resin and mercaptan repaired the polymeric matrix, while a coordination bond was established between the sulfhydryl group of the mercaptan and the silver nanoparticles, contributing to the regeneration of the fiber/matrix interface. In addition, the healing reaction between the epoxy resin and the mercaptan is strongly exothermic; therefore, thermal signals were released from the crack healing, which were then captured by an infrared thermal imager; thus, the damage and the healing reaction could be monitored. A healing efficiency of 80% was attained after 24 h, at room temperature, for a microcapsule content of 15 wt%, evaluated by fracture toughness. Moreover, the healing effectiveness at the interface, analyzed resorting to the interface shear strength, reached 74% for the same microcapsule content.

Nevertheless microcapsules have some limitations, particularly with regard to the amount of repairing agent they contain, making it impossible to know when it has been completely consumed or to feed it back into the system, as the number of capsules is finite. In fact, that optimum amount of microcapsules is typically determined for each prepared composite by analyzing the impact of varying mass percentages of incorporated microcapsules on the healing efficiency, the loss of original mechanical properties, as well as other material properties (dynamic mechanical analysis (DMA); differential scanning calorimetry (DSC); thermogravimetric analysis (TGA)). Through this method, it is possible to identify the point at which increasing this percentage no longer significantly increases the healing efficiency and may begin to negatively affect the aforementioned mechanical properties. Alternatively, numerical simulation can be used to predict this behavior [3,10,19,36,43,44]. Other effects that must be considered include the probability of microfractures meeting the capsules randomly dispersed in the matrix, leading the repair agent to be released; the material, size distribution and thickness of these capsules; the aforementioned compatibility with the matrix, the hypothesis of particle coalescence and the influence that these characteristics have on the properties of the matrix itself. Furthermore, after the capsules break and the regenerating compound is released, the empty capsule remains in the matrix, enabling the accumulation of tension at the site, which could have negative consequences, especially in the case of structural composites, ultimately leading to the re-occurrence of damage in the same location. However, this behavior is still scarcely studied. In addition, after the second impact, the failures must follow a path that ensures that the crack meets the unbroken microcapsules; otherwise, the damage might not be healed, increasing the possibility of microcrack expansion into more complicated macro-damage [22,29,37,44]. As an alternative, scientists have been putting effort on developing microcapsules with the ability to repeatably self-heal, by imparting them with multi-storage vessels. In other words, a single microcapsule could independently accommodate more than one portion of healing agent [26,45,46]. For that purpose, Ying Xue et al. [26] developed “hierarchical microcapsules with multi-storage cells” that were incorporated into coatings. They concluded that, in fact, such coatings exhibited multiple self-healing abilities in the same area, because, during the first damage, the central cell would rupture, releasing the functional compounds, whereas during the following damage, microcapsule-type stabilizers attached to the surface of the central cell would rupture, releasing the second portion of healing agent.

Furthermore, microcapsules may not be suitable for composites that are manufactured under high pressure considering that their shape can be deformed [22,29,37]. Urea-formaldehyde, melamine-urea-formaldehyde and polyurethane capsules are preferred, considering that they exhibit mechanical resistance so as not to be degraded during processing, but are still soft enough to rupture relatively quickly when necessary [4,29]. Commonly, in situ or interfacial polymerization or generally methods that resort to emulsion or melt dispersion are used, although other techniques, like electrospraying, have also been tested. In the first group, the core content of the microcapsules is dispersed in a solution of the shell polymer’s components or vice versa. Initially, droplets are formed and then the polymeric layer is synthesized and solidified around them. There are some parameters that are determinant for the morphology and size of the microcapsules, that, in turn, influence the composite’s characteristics and these include the stirring rate, temperature, reaction time, weight ratio of core/shell material and emulsifier content, if applicable. For instance, smaller microcapsules with a thin surface and no evidence of collapse stand out for promoting fewer negative impacts on the composite’s mechanical properties and can even endorse extra strengthening, as they possess smaller tendency to agglomerate, reduce the stress concentration and cause less reinforcing fiber misorientation. Even so, larger microcapsules can be associated with a higher healing efficiency, particularly in the case of lower volume fractions (<15%), as they are synonymous with a superior quantity of healing agents as well a tendency to rupture more easily. With that in mind, in each case, it is necessary to determine the optimal capsule size that balances both healing efficiency and a reduction in original mechanical properties [11,15,22,25,28,29,36,43,44,47,48,49,50].

Furthermore, it may also be pertinent to investigate the utilization of hybrid microcapsules of different dimensions (nanometric and micrometric) as these could potentially address damage at different scales and enable a controlled healing mechanism, together with the usage of healing agents with distinct reaction times. Specifically, smaller capsules could quickly release fast-reacting healing agents to address immediate minor damages, while bigger ones would release larger quantities of healing agents, targeting larger damages and possibly releasing slow-reacting compounds gradually, thus contributing to long-term healing. Additionally, the combination of larger and smaller microcapsules could contribute to achieving a better balance between healing efficiency and conservation of original mechanical properties [25,49].

**Table 1:** Self-healing through capsules.

Composite	Healing Agents	Healing Conditions	Healing Efficiency	Other Notable Findings	Reference
Epoxy matrix composite	Two types of microcapsules containing: - Same polymer as the matrix (12 wt%) - Amine curing agent (8 wt%)	24 h at room temperature, followed by 6 h at 50 °C	80.4% (Fracture toughness)		H. Hu et al. [36]
Epoxy matrix composite	Two types of microcapsules containing: - Styrene (15 wt%) - Benzoyl peroxide (3 wt%)	24 h at 25 °C	65% (Impact tests)	Multi-repairability reduced efficiency by 20% to 30% after second and third repairs	C. Y. Zhang et al. [37]
Epoxy matrix composite	- Microcapsules containing: monomers of 5-ethylidene-2-norbornene with multi-walled carbon nanotubes (10 wt%) - Matrix dispersed Ruthenium Grubbs catalyst	48 h at room temperature	97% (Fracture toughness)	Carbon nanotubes improved healing efficiency	H. H. Zamal et al. [38]
Epoxy matrix composite	Microcapsules containing epoxy resin inside and curing initiator catalyst (Scandium triflate (III)) on the outer surface (20 wt%)	24 h at 120 °C	79.1% (Fracture toughness)	Microcapsule structure improved reaction probability between resin and catalyst	R. Rodriguez et al. [39]
Epoxy-based foams	Multi-core-like bilayer calcium-alginate capsules (15% vol.) containing: - Epoxy resin on the inner core - Curing agent on the outer core		>80% in the first cycle (Elastic modulus)	Multi-core capsules provided a better mixing of the healing agents, increasing performance	Shunze Cao et al. [23]
Carbon fiber/epoxy composite	Microcapsules containing an epoxy resin-solvent solution	24 h at room temperature	91% (Interfacial shear strength)	Usage of a binder stabilized the microcapsules on the fibers’ surface	Amanda R. Jones et al. [41]
Polymer matrix with carbon fibers	- Microcapsules containing an epoxy resin, mercaptan (15 wt%) - Modified silver nanoparticles on the surface of the reinforcing fibers	24 h at room temperature	80% (Fracture toughness) 74% (Interface shear strength)	Coordination bond between mercaptan and silver nanoparticles regenerated fiber/matrix interface	Wrihao Yuan et al. [42]

#### 2.2.2. Hollow Fibers

**Figure 4:**
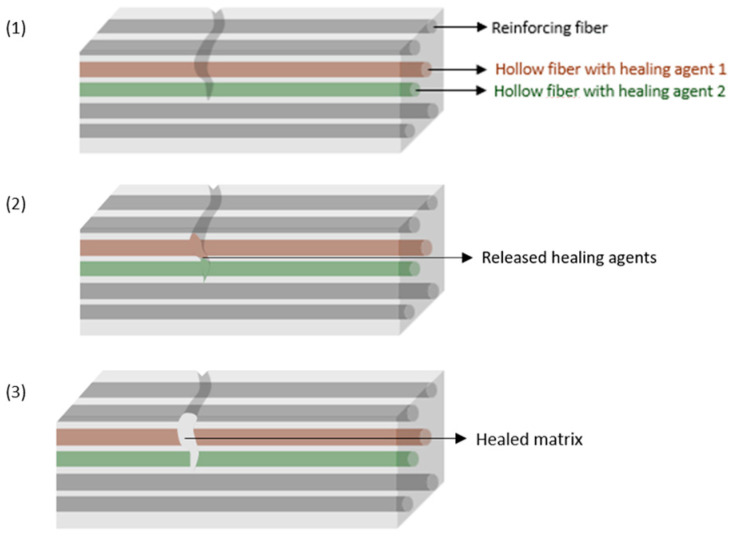
Scheme of a composite consisting of hollow fibers containing the healing agents and reinforcing fibers: (**1**) fracture formation; (**2**) release of repair agents contained in the hollow fibers; (**3**) reparation of the matrix.

For hollow fibers (Figure 4 [4]) (Table 2), their operating principle is similar to that of capsules; however, they allow to overcome some of the latter’s limitations, particularly with regard to storing larger volumes of healing agent and transporting them to greater distances. It is also worth noting that the fibers may cover a greater volume of the material, increasing the probability of the reservoir breaking when the fracture occurs, and can be arranged on demand, contributing to a more uniform distribution of the healing agents through the final material, even benefiting the stoichiometric mixture of the referred reactants. These fibers can be incorporated directly between the reinforcing fibers. Similarly to the microcapsules, two healing agents can circulate through different fibers, as shown in Figure 4 [1,4,11,29].

In this setting, A. Adili et al. [51] developed a healing system combining an epoxy resin and an amine curing agent, both equal to the matrix’s materials and each one deposited inside a different glass fiber. It was observed that, when empty, the glass fibers negatively affected the material’s strength due to stress concentration and crack initiation. Despite that, it was concluded that smaller distances between the fibers were indicative of a higher amount of healing agent and, therefore, resulted in an improved healing performance. Additionally, when positioned in angles of 45 º, the fibers underwent higher shear stress and consequently were more prone to fracture, leading to a wider diffusion of the healing agents and contributing to a better healing efficiency, which was 42%, determined by tensile strength, after 48 h at 70 °C. In contrast, S. Kling [52] proposed a healing response based on a polyester resin system stored inside thin hollow glass fibers, with an outer diameter of 13 µm, and integrated into an epoxy matrix composite. One type of fiber was filled with the catalyzed unsaturated polyester resin and the other one contained the initiator (methyl ethyl ketone peroxide) dissolved in dimethyl phthalate. This option was justified by the fact that this reacting system is not as sensitive to the mixing ratio and method as others. It was demonstrated that the composite was self-healable at 23 °C over 120 h. A method based on the combined answer of a monomer and catalyst can also be applied to these structures, as I. Radovic et al. [32] proposed. Here, the monomer dicyclopentadiene was mixed with a solvent (N, N′-dimethylformamide) and fed to one type of fiber, while the first-generation Grubbs catalyst, also dissolved in a solvent (preferably toluene, since other solvents such as dichloromethane can deactivate the catalyst during processing), was fed to another type. In this case, it was found that, when filled with the healing components, the fibers actually operated as reinforcing agents, and the healing efficiency, determined through impact tests, achieved a value of 53%, after 24 h at 25 °C.

On a different approach, S. A. M. Sadeghi et al. [53] developed a core–shell nanofiber mat resorting to single-nozzle electrospinning, where epoxy resin and a mercaptan-based epoxy hardener were enclosed. This mat was then incorporated into an epoxy-resin-based composite, and it was noted that the presence of the healing agent on the surface of the nanofibers promoted a good adhesion between the matrix and the mat. The conclusion was that the material was able to self-heal and restore flexural modulus and strength after being kept at 10 °C for 200 min.

A groundbreaking strategy was developed by Y. Zhu et al. [54], where the mixing of the healing agents with a foaming agent was evaluated, together with the utilization of polypropylene tubes. These tubes were chosen, considering that they allow for the healing agents to be easily filled into larger tubes whose diameter can then be reduced, overcoming not only the difficulty of filling narrow tubes, but also preventing the weakening of the mechanical properties caused by larger tubes. In addition, the tubes’ flexibility allows for their knitting together with the traditional reinforcing fibers, in this case, glass fibers, which is beneficial for materials with complicated shapes. Although polypropylene’s natural non-polarity can compromise interfacial adhesion, there is the possibility of modifying its physicochemical properties in order to improve such interaction. Finally, this polymer does not tend to react with the healing compounds that it contains. Nevertheless, the polypropylene tubes had to be irradiated by UV light with the aim of embrittling and polarizing the surface so that they would break upon damage of the composite. The healing response relied on an epoxy resin cured by a thiol and amine mixture, and 2,2′-Azobis-(2,4-dimethylvaleronitrile) was added as the foaming agent, which does not affect the healing agents’ cure. The material was previously pressurized so that when damage occurred, the high internal pressure of the foaming agent favored the spreading of the healing reactants over the damages. The healing efficiency, determined through flexural strength of the healed specimen in comparison to the virgin one, was about 93%, but a decrease in such parameter was identified when the storage time increased, due to leakage of the pressurized foaming agent, therefore suggesting that polypropylene impermeability could be improved.

At this point, it can be highlighted that the main challenge of this approach is the filling of the hollow fibers with the functional components due to their reduced diameter. Some techniques include filling from the top or drawing from the bottom, and typically they are achieved by capillary action, vacuum assistance or even through surface pores that are promptly covered after infusion. To aid the filling process, the mixing of the healing agents with solvents can also be useful. Emerging techniques can include electrospinning or fibers whose diameter can be adjusted post-filling. In addition, if the diameter of the fibers is too small, the viscosity of the regenerating agent can limit its flowability and, therefore, transport to the damaged area. However, larger diameters are more prone to contribute to the degradation of the materials’ mechanical properties. For that reason, technical issues related to using these structures can include the production of hollow fibers with small and uniform diameters, as well as relative structural integrity, which are still cost-effective [1,22,28,32,51,52,53,54]. As shown, the utilization of glass fibers is typically reported, as they break at lower energy levels, and it is conjectured that they can act not only as a transport route for the repairing agents in question, but also as a structural reinforcement of the composite material [1,4,24]. The application of carbon nanotubes is also suggested; however, it is assumed that their high resistance may not allow for breakage during fracture formation [27]. In addition, the amount and spatial distribution of the fibers should also be scrutinized due to their effects on the mechanical behavior and healing response of the composite, as some of the previously mentioned articles highlight. For instance, increasing the space between hollow fibers can diminish the impact on the mechanical properties; however, healing performance can be compromised as well [24,28].

#### 2.2.3. Microvascular Networks

**Figure 5:**
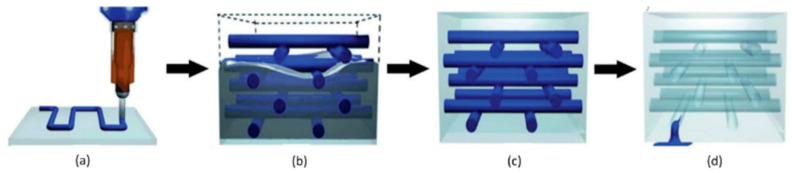
Manufacturing process of a self-healing composite material consisting of a capillary network: (**a**) Deposition of the non-permanent material to develop the network of capillaries, (**b**) deposition of the matrix material, (**c**) solidification of the material, (**d**) removal of the non-permanent material and final shape of the composite with the network of capillaries. Adapted from “Progress and challenges in self-healing composite materials” by Shafiqul Islam and Gajanan Bhat, used under CC BY 3.0. [https://doi.org/10.1039/D0MA00873G] [1].

To overcome the limitations associated with capsules and hollow fibers, there has been growing interest in studying the distribution of the healing agents through capillary networks which, as the name suggests, consist of 2D or 3D networks of interconnected microchannels that transport the compounds in question to the damaged areas (Table 3). This method is, in part, similar to the previous one; however, it differs in its network of interconnected channels. These, unlike individual hollow fibers, make it possible to continuously feed the damaged site, allowing more than one repair event to be carried out in the same location, which is complemented by the fact that the system can be refilled. Ultimately, this feature can be enough to enhance the repair efficiency of the materials. Hence, a fresh healing agent can be supplied, preventing it from being stored for too long and eventually losing its activity. In this context, the hypothesis of integrating reservoirs capable of automatically detecting the need for repair and consequently feeding the healing agent into the system has already been considered [1,11,27,29]. With this model, it is also possible to add the repairing compounds in distinct types of capillaries. This perspective is, therefore, considered one of the most promising ones in the field of auto repair materials [1,3]. Nevertheless, the development and practical application of this concept is difficult, especially in large-scale materials, considering the complexity of the used methods of manufacturing that can consequently be associated with higher production costs. Capillary networks can be developed based on non-removable or removable preforms. In the first case, like the other types of reservoirs, the capillaries must be broken so that the repairing reactants are released in the fracture, leading to concerns already mentioned. In the second case, the structure of the material intrinsically integrates the hollow capillary network where the repaired agents circulate, meaning that they can flow immediately to the damage, when it is formed, without the need for reservoir breakage. However, this last option poses some limitations. On the one hand, the compounds circulating in the network are not protected, being in direct contact with the matrix, and, on the other hand, there is the possibility of the channels’ deformation during the manufacturing process [11,29,55]. The proposals for the development of removable preforms comprise wires coated with a release agent that allows for the manual extraction of such wires, or 3D printing using a non-permanent material/fugitive organic ink made up of a mixture of high- and low-molecular-weight hydrocarbons, for example, a mixture of 60% petroleum jelly and 40% microcrystalline wax. The vascular network is developed at an initial stage and only then is the material itself formed, as shown in Figure 5. The mentioned fugitive ink is removed after the matrix resin has cured, by heating at a moderate temperature, under light vacuum, so that it is liquefied. In this way, the final material is left with space in channels than can be filled with healing agents [1,22,30,56,57].

On that matter, R. Eslami-Farsani et al. [58] designed a microvascular network resorting to a digital light-processing method, based on the Vat polymerization technique and using a 3D printer. Such a network was made of two different sub-networks: one where the epoxy resin flowed (the same as the matrix) and another with the curing agent. Besides the microvascular system, the composite was also reinforced with glass fiber. It was concluded that the network presence led to a reduction in the composite’s tensile strength; however, it was also suggested that such a problem could be overcome by selecting a more rigid material for the network. A healing efficiency of 89% was calculated by resorting to tensile strength after 7 days at ambient conditions. Moreover, R. S. Amano et al. [59] imprinted a vascular network on one layer of glass-fiber-reinforced composite. The vascular network was filled with dicyclopentadiene, while the Grubbs catalyst was infused in this same layer; such a system was responsible for the self-healing response. Two patterns for the vascular networks were analyzed: square and hexagonal. It was concluded that the average strength of the composite, attained via a three-point bending test, nearly doubled after recovery, which is attributed to the reaction of the healing agents and the formation of polydicyclopentadiene, which is about 50% as strong as the matrix’s epoxy resin. Additionally, it was observed that a hexagonal grid favors access to the contained healing agent during damage formation. The final purpose of these developed materials was to produce self-healing wind turbine blades.

O. Fifo et al. [30] proposed an alternative approach where an in vivo vascular channel was employed for the self-healing of a glass fiber polyester composite. It preferably resorted to an automated process of injection of a single-part adhesive (cyanoacrylate adhesive) into a 2D network of hollow channels directly integrated within the composite. Such a network was built resorting to a nylon cord coated with wax that was removed at the end of preparation. This network was located in the material’s mid-plane, so that damages originating from either surface could contact the hollow channels. An average recovery of 84% of the flexural stiffness and 46% of the loading strength was determined, based on bending tests, after self-healing at ambient conditions, for 24 h. Furthermore, J. F. Patrick et al. [56] resorted to sacrificial poly(lactic acid) monofilaments arranged in two different patterns through the woven fabric of an epoxy-resin-based fiber-reinforced composite that were removed after the composite’s cure. The healing answer was based on the action of an epoxy-resin- and an amine-based curing agent, both of low viscosity. The first configuration was parallel, with the mixing of the two components being limited to adjacent layers, while the second one was a herringbone design, where the blending of the healing components was favored by the overlapping of the networks. For that reason, it was concluded that the herringbone design provided higher healing efficiencies for specimens healed at 30 °C for 48 h. Additionally, over 100% healing efficiency was observed due to the higher fracture toughness of the healed polymer, when compared to the structural matrix. Three successive healing cycles were performed and after each one, higher loads were necessary for the crack to propagate.

Relevant parameters for the microvascular networks include the microchannels’ dimensions, as well as the network feeding mode, the pressure of the vascular system and the viscosity of the repairing agent, to ensure that it is properly distributed. Therefore, if necessary, pumps can be incorporated outside to direct the flow of the repair agent to the damaged areas [1,22,27].

There is also the possibility of combining the aforementioned approaches based on reservoirs in order to take advantage of the benefits that each one provides. For example, the resin can be inside hollow fibers, while the corresponding curing agent is microencapsulated [60].

### 2.3. Self-Healing through Thermoplastic Polymers

**Figure 6:**
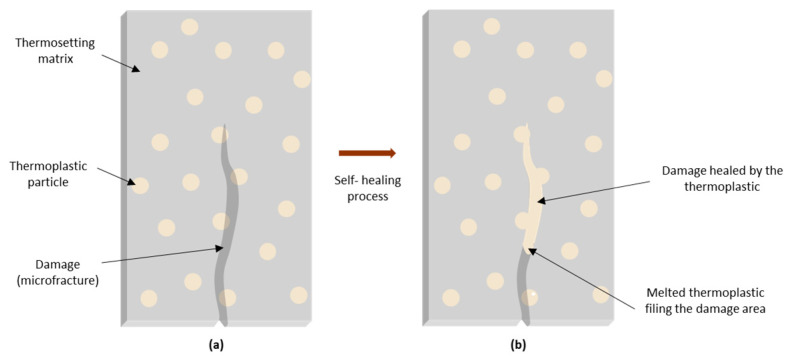
Healing process using thermoplastic particles: (**a**) fracture formation; (**b**) melting of the thermoplastic polymer, followed by cooling and consequent matrix reparation.

In the case of self-healing using thermoplastic polymers (Table 4), these are incorporated into the matrix, for example, in the shape of particles, films or fibers, either mixed with the thermosetting resin or incorporated between the carbon fiber plies, prior to resin impregnation. Upon heating and consequent melting, the thermoplastic migrates by diffusion to the flaws when they arise, and establishes chemical bonds with their surfaces, as schematized in Figure 6. Accordingly, a good affinity between the thermoplastic and the commonly thermosetting matrix is required so that an efficient healing occurs and there is stress transfer between the thermoplastic and the epoxy matrix. Some of the thermoplastic polymers already investigated for this purpose include poly(ethylene co methacrylic acid) (EMAA), poly(methyl methacrylate) (PMMA), ethylene vinyl acetate (EVA), poly(ethylene co glycidyl)-methacrylate (PEGMA), poly(vinyl butyral) (PVB), styrene ethylene butadiene copolymer (SEBS) and acrylonitrile-butadiene-styrene (ABS). In addition to these factors, some authors [61,62] also highlight the role of small bubbles present inside the thermoplastic, particularly EMAA, that expand upon heating, generating a high internal pressure difference that will force the thermoplastic into the crack, imposing the polymer flow. Such bubbles are theorized to be formed as a result of chemical reactions taking place between the thermosetting matrix and the thermoplastic polymer containing carboxylic acid groups on their structure, which produce water as a volatile product whose solubility in the polymer is very limited. Such reactions might include condensation between the hydroxyl groups of the epoxy matrix and the carboxylic acid groups of the filling polymer, or amidation of these carboxylic groups. One of the main disadvantages of the present alternative is that it requires heat for the melting and diffusion of the repairing polymer to occur, meaning it is not a completely autonomous system. However, it theoretically allows for unlimited cycles of self-healing since it can be repetitively melted and solidified [4,15,20,29,63,64].

Within this framework, A. Azevedo et al. [64] incorporated poly(ethylene-co-methacrylic acid) microparticles (5 wt%) between prepreg layers at the mid-plane of carbon-epoxy laminates. The incorporated thermoplastic established chemical interactions with the epoxy matrix, promoting a strong interfacial adhesion what, in turn, contributed to a complete recovery of the interlaminar shear strength, after healing at 150 °C during 30 min. Nevertheless, the addition of poly(ethylene-co-methacrylic acid) promoted a slight increase in the glass transition temperature and a decrease in the composite’s interlaminar shear strength and storage modulus when compared to the virgin material. Differently, M. Peñas-Caballero et al. [63] developed an innovative approach where poly(methyl methacrylate) was applied, as a healing agent, to reinforcing carbon fibers, by spray coating, and directly mixed with the epoxy-based matrix, amounting for 20 wt% PMMA. It was concluded that the thermoplastic polymer interacted with the epoxy thermosetting polymer through a transesterification reaction. However, trials with a zinc acetate catalyst led to the observation that such an interaction was not the driving mechanism for the healing process, which was instead led by the softening and flow of the polymer. Nevertheless, the reaction still improved the interface adhesion and was therefore favored by the catalyst action. Furthermore, the fibers were uniformly coated, but some reduction in the mechanical properties was observed. Despite that, the composites exhibited up to 53% healing efficiency, determined by the fracture toughness, after heating at 150 °C, for 120 min.

Another breakthrough method was proposed by B. Chen et al. [65], where polyamine nanofibers where electrospinned and deposited on the surface of a unidirectional carbon fiber fabric. This thermoplastic polymer was uniformly distributed between the interlayers of a carbon-reinforced epoxy composite. The main objective was that the polyamine entangled fibers worked both as a toughening material for the interlayer of the composite and as a self-healing agent. The addition of 1.2 wt% of the thermoplastic polymer increased the interlaminar shear strength by 17.6% and the bending properties by 14.7% due to the large specific surface area and the good interaction between the polyamide and the epoxy matrix, which improved interface adhesion. The healing process took place at 130 °C, over 20 min, and the efficiency was analyzed throughout three cycles, resorting to the interlaminar shear strength. A maximum value of 110.44% was determined for the first one, for an electrospinning time of 3 h. It was also highlighted that the incorporated fibers did not affect the storage modulus, nor the glass transition temperature of the composites. Innovatively, R. B. Ladani et al. [62,66] developed a carbon-fiber-reinforced epoxy composite designed to resist the growth of delamination cracks and self-heal such flaws. For that purpose, they resorted to through-the-thickness z-binders, namely, one type made of carbon fiber, to achieve the first goal, and the second one made of thermoplastic filaments, particularly poly(ethylene-co-methacrylic acid), to achieve the second one. The hybrid composite underwent an increase both in the mode I fracture toughness (1200%) and in the mode II fracture toughness (75%). Self-healing was then stimulated at 150 °C for 30 min. A recovery of 35–40% of the original value of the mode I fracture toughness was observed, whilst a restoration of 25% of the original mode II fracture toughness was measured. The significant difference between the two cases is justified by the fact that the molten thermoplastic polymer could more easily flow into the mode I wider delamination crack.

The temperature dependence could be overcome through the utilization of an encapsulated solvent that, during the formation of the fracture, would be released, dissolving the thermoplastic, transporting it to the necessary area and evaporating at the end, in a somewhat similar approach to the previously described capsules [31,67]. Another alternative would be to induce localized fusion, using superparamagnetic nanoparticles, that is, nanoparticles that only exhibit magnetization when subjected to an external magnetic field, embedded in thermoplastic. Thus, upon the application of such a field, the temperature of the nanoparticle/polymer interface would increase, triggering the melting of the thermoplastic that would flow towards the failure. Graphene can also lead to localized heating when exposed to electricity, infrared radiation or electromagnetic waves; so, the application of a principle similar to the previous one is also a hypothesis [27].

In light of this, K. M. Chang [67] developed a composite where a thermoplastic resin, poly(bisphenol A-co-epichlorohydrin), was dispersed on a carbon-fiber-reinforced epoxy matrix together with an encapsulated solvent (ethyl phenyl acetate). The healing efficiency of such a system was compared with a traditional one, where temperature was used to melt the thermoplastic resin instead, and the conclusion was that no statistically significant differences existed between the two approaches. That meant that the usage of solvents was as feasible as the conventional method and the healing efficiency determined through tensile tests was 57%.

As explained in the reservoir-based approach, here, it is also important to address the consequences of thermoplastic incorporation on the composites’ mechanical properties, considering that strength reduction might occur. For that reason, there are limitations regarding the dimensions of the incorporated particles; nevertheless, it is suggested that thermoplastic repairing agents are efficient and easier to prepare and incorporate into the matrix when compared to the capsule and fiber systems discussed in the previous paragraphs [4,15,29].

Throughout this review, it becomes clear that providing a composite with self-healing capacity comes at the expense of some of the materials’ properties, mostly mechanical ones. For that reason, the choice of an adequate approach to self-healing will depend on the nature of the materials involved, its applications, the adequacy of a certain stimulus and the trade-off between the properties’ loss and the necessary healing efficiency.

## 3. Applications

Despite being suitable to repair essentially small damages and, in some cases, prevent further damage, self-healing composites can still be implemented in varied areas, especially standing out in applications with difficult access and high inspection and maintenance costs, such as wind turbine blades. At the moment, the most attractive fields are probably the aerospace and aeronautic industries, particularly for structural parts, for example, wings, which must withstand high temperatures and pressures and resist impact, fatigue, corrosion and environmental factors. In fact, the European Space Agency has considered self-healing materials “one of the most futuristic concepts” for spacecraft due to the extreme fluctuations in the environmental conditions and the high-velocity impacts that these can undergo. As a matter of fact, it is estimated by this agency that the materialization of this principle could double the lifetime of an Earth-orbiting spacecraft, therefore halving the overall cost of the mission [5,10,11,12,15,16,20,38,68,69]. Also, in the automotive industry, these composites might find applications in vehicle body parts, bumpers, tires and even batteries that can recover from damage, such as dents, puncture damage and wear, thus maintaining appearance, functionality and performance. In such a way, the materials in question contribute to improving the safety, efficiency, lifespan and even the environmental impact of vehicles, minimizing the need for replacements or repairs. Another example involves civil engineering structures, such as buildings or even pipes, as self-healing capacity would allow them to resist not only damage and deterioration, but also natural disasters, such as earthquakes, floods or fires. In this way, the safety, integrity, durability and resilience of infrastructures would also be improved, reducing repair costs. In the same line of thought, their employment in defense should also be considered, as they can contribute to a wider lifespan of military equipment and improve itssecurity. On a different approach, these materials can be used in biomedicine to produce implants, prostheses, dental composites and sutures, which can resist the biological environment, recovering from damage caused, for example, by infection or inflammation. This would improve the functionality, effectiveness and durability of biomedical devices, reducing possible complications associated with side effects. Additionally, self-healing composites have also been considered for coatings, as they can repair damages and prevent failure as a consequence of corrosion [11,15,20,22,44,69].

## 4. Challenges, Opportunities and Future Prospects

In light of this review, interest in self-healing composites with polymeric matrices has indeed been growing in recent years and the pros and cons of each technique are summarized in Table 5. However, such technology is yet to be scaled up for practical applications, as the repair efficiency can vary significantly depending on damage size and the environmental conditions, such as pressure and temperature, at which the material is used, affecting, for example, the viscosity of the repair agent. Additionally, self-healing time is also a very important parameter, considering that it is crucial to avoid the catastrophic failure of a composite that is being applied in practice. This variable is dependent not only on the extent of damage but also on the healing mechanism and environmental conditions. For that reason, healing efficiency is deeply influenced by healing time [11,69,70]. It is also important to note that over time, healing agents can lose their reactivity, causing the material to gradually lose its self-repairing capacity. Both the storage and aging of healing agents are critical for ensuring long-term performance of self-healing composites. During storage, issues like evaporation and pressure build-up, due to temperature and pressure fluctuations, become particularly critical, especially in extreme environments such as the already mentioned wind turbines and aerospace applications. For healing systems relying on reservoirs, encapsulation technologies must be robust and low-volatile solvents should be preferred to prevent loss of functionality. When it comes to aging, factors like temperature, moisture absorption, oxidative degradation, and UV exposure can further degrade the healing agents. To mitigate these effects, barrier properties of reservoirs can be improved, and stabilizers included to delay degradation. Ultimately, thorough testing and monitoring under real operational conditions will be essential to successfully implement this technology. In addition, the efficiency of this capacity tends to reduce as multiple failure and repair cycles occur. Moreover, although through numerical simulations a prediction can be made of where the material is going to fail, it is not completely accurate and therefore it is not possible to forecast where the self-healing system should be specifically introduced. Furthermore, and as previously discussed, it is very important to take into account that functionalization with self-repairing agents can trigger significant changes in the morphology and performance of the material and, consequently, in its physical and mechanical properties. It is also essential to consider that a sufficient amount of energy is necessary for these structures to break, since if this energy is dissipated before reaching the reservoirs, the damage will not be repaired [1,4,8,20,27,29,30]. Aside from that, the studied systems that make use of catalysts are unaffordable in realistic practical applications, considering the high price and toxicity of catalysts [23,71]. Furthermore, when talking about cost, and as pointed out at the beginning of this review, studies indicate a possible cost reduction associated with less maintenance and extended lifetime. However, the cost of production is typically higher than that of common materials. This analysis is therefore complex and context-dependent in terms of the used mechanism, application and scale of implementation. A balance between both initial costs associated with manufacturing and long-term savings related to reduced maintenance and longer service life should be achieved [11,15,69,72,73,74]. For these materials to be applied in practice, such limitations must be thoroughly studied and overcome.

In addition, the self-healing activity can still be innovated upon, for example, through combination with other functionalities, including shape memory or stimulus responsiveness, like pH, temperature or electric field. Another interesting concept is the combination of repair activity with the signalization of this same action, for example, through the presence of a fluorescent compound or through the thermal monitoring of the healing reaction [42,75]. This principle would be particularly interesting for applications of difficult to access, since, on the one hand, the self-repairing action allows the damage caused to be reversed, to a certain extent and, on the other hand, the signalization of the repair action allows for the identification of the area where the damage and reparation occurred.

With this in mind, it can be said that the path for self-healing mechanism to redefine materials’ resilience is at a stage where current knowledge needs further refinement. This is particularly true in two areas: first, exploring breakthrough approaches that have not yet been thoroughly investigated, and second, transferring these advancements into practical applications, addressing scalability and integration into real-world products adapted to various environments and conditions. Additionally, enhancing the autonomy of self-healing systems to meet future needs is crucial. These steps are essential for advancing the future of material resilience [11,28].

## 5. Conclusions

This review has provided an insight into extrinsic self-healing mechanisms for polymer composites, highlighting their autonomy and applicability to different types of materials. The conclusions are the following:

- Two general approaches exist for preparing self-healing composites, namely, through the incorporation of either reservoirs containing the healing agents or thermoplastics.
- Among the reservoir-based approaches, capsules are the most extensively investigated due to their ease of application and the fact that they are the focus of groundbreaking research aimed at increasing their efficiency.
- Hollow fibers and microvascular networks are emerging as promising options due to their wider coverage of the materials’ volume and the ability to be refilled. Such reservoirs are also under intense study, with alternatives emerging to facilitate their integration into the composites’ structure.
- Thermoplastics have also been widely explored, particularly considering their theoretically unlimited healing cycles. However, research is committed to developing alternative polymer melting mechanisms that allow for the complete independence of the healing process.
- Globally, all the methods tend to jeopardize mechanical properties, requiring a balance between this loss and the self-healing ability.

In summary, when considering the selection of a particular self-healing strategy for scaling at an industrial level, thermoplastic-based approaches stand out considering the associated simplicity and compatibility with existing manufacturing processes. However, research indicates that further work is still needed to address parameters that are crucial for transferring self-healing into practical applications, including healing efficiency and the autonomy of the systems. Finally, further innovation continues to be essential to ensure that this technology is explored to its full potential.

## Figures and Tables

**Table 2:** Self-healing through hollow fibers.

Composite	Healing Agents	Healing Conditions	Healing Efficiency	Other Notable Findings	Reference
Epoxy resin matrix with glass fibers	Two types of glass fibers containing: - Epoxy resin - Amine curing agent	48 h at 70 °C	42% (Tensile strength)	Smaller fiber distances and angled fibers (45° increased efficiency	A. Adili et al. [51]
Epoxy resin matrix with glass fibers	Two types of glass fibers containing: - Unsaturated polyester resin - Methyl ethyl ketone peroxide initiator, dissolved in dimethyl phthalate	120 h at 23 °C			S. Kling [52]
Epoxy resin matrix with hollow fibers	Two types of hollow fibers containing: - Dicyclopentadiene, dissolved in N, N′-dimethylformamide - First-gen Grubbs catalyst dissolved in toluene	24 h at 25 °C	53%, (Impact tests)	Filled fibers acted as reinforcing agents	I. Radovic et al. [32]
Epoxy matrix composite	Core–shell nanofibers mat containing: - Epoxy resin - Mercaptan-based hardener	200 min at 10 °C	The material was able to self-heal and restore flexural modulus and strength	Good adhesion between matrix and nanofiber mat	S. A. M. Sadeghi et al. [53]
Epoxy composite with glass fibers and polypropylene (PP) tubes	PP tubes containing: - Epoxy resin - Thiol and amine mixture + foaming agent		93% (Flexural strength)	PP tubes contributed to overcome filling difficulties. PP tubes were UV - irradiated to break upon damage	Y. Zhu et al. [54]

**Table 3:** Self-healing through microvascular networks.

Composite	Healing Agents	Healing Conditions	Healing Efficiency	Other Notable Findings	Reference
Epoxy resin matrix with glass fibers	Two subnetworks containing: - Epoxy resin - Curing agent	7 days at ambient conditions	89% (Tensile strength)	Microvascular network reduces tensile strength but could be improved with a more rigid material	R. Eslami-Farsani et al. [58]
Epoxy resin matrix with glass fibers	Network filled with dicyclopentadiene Matrix dispersed Grubbs catalyst		Nearly doubled strength after recovery (Three-point bending test)	Hexagonal grid favors healing agent access	R. S. Amano et al. [59]
Polyester matrix with glass fibers	Two-dimensional vascular network containing single-part cyanoacrylate adhesive	24 h at ambient conditions	84% (Flexural stiffness) 46% (Loading strength)	Channels located in mid-plane for dual-surface damage access	O. Fifo et al. [30]
Epoxy matrix with fiber reinforcement	Network containing: -Epoxy resin - Amine-based curing agent (low viscosity)	48 h at 30 °C	Over 100% due to higher fracture toughness of healed polymer	Two patterns (parallel and herringbone) analyzed: herringbone pattern showed better efficiency and toughness; Three successive healing cycles increased load resistance	J. F. Patrick et al. [56]

**Table 4:** Self-healing through thermoplastic polymers.

Composite	Healing Agents	Healing Conditions	Healing Efficiency	Other Notable Findings	Reference
Epoxy matrix with carbon fiber reinforcement	Poly(ethylene-co-methacrylic acid) microparticles (5 wt%)	150 °C for 30 min	Complete recovery of interlaminar shear strength	Slight increase in glass transition temperature but decrease in interlaminar shear strength and storage modulus	A. Azevedo et al. [64]
Epoxy matrix with carbon fiber reinforcement	Poly(methyl methacrylate) (20 wt%)	150 °C for 120 min	53% (Fracture toughness)	Softening and flow of polymer drives healing; Zinc acetate catalyst enhances interface adhesion but reduces mechanical properties	M. Peñas-Caballero et al. [63]
Epoxy matrix with carbon fiber reinforcement	Electrospun polyamine nanofibers (1.2 wt%)	130 °C for 20 min (3 cycles	110.44% (Interlaminar shear strength recovery for first cycle)	Improved interfacial adhesion and bending properties without affecting storage modulus or glass transition temperature	B. Chen et al. [65]
Epoxy matrix with carbon fiber reinforcement	Through-the-thickness Z-binders: - Carbon fiber - Poly(ethylene-co-methacrylic acid))	150 °C for 30 min	35–40% (Mode I fracture toughness) 25% (Mode II fracture toughness)	Hybrid composite improves mode I and II fracture toughness (1200% and 75%, respectively)	R. B. Ladani et al. [62,66]
Epoxy matrix with carbon fiber reinforcement	Matrix dispersed poly(bisphenol A-co-epichlorohydrin) resin-encapsulated solvent		57% (Tensile tests)	No significant differences between solvent-based and temperature-based methods for healing	K. M. Chang [67]

**Table 5:** Pros and cons of self-healing techniques.

Self-Healing Mechanism	Pros	Cons
Capsules	- Simple and well-studied - Effective for small, localized damage	- Typically one-time healing only - Limited healing agent storage - Distribution and uniformity of capsules can be uneven, limiting efficiency - Presence of empty microcapsules after damage can compromise material’s integrity
Hollow fibers	- Larger storage capacity for healing agents compared to capsules - Can provide healing over a larger area - Controlled release of healing agents upon fiber rupture	- Still tendentially a one-time healing system - Fibers may compromise materials’ structural integrity - Difficult to repair fibers once damaged, limiting repeated healing
Microvascular Networks	- Continuous supply of healing agents, allowing for multiple healing events - Can provide healing to larger, more complex areas - More durable than capsules or fibers	- Complex fabrication and integration into composite materials - Prone to clogging or blockage, which can disrupt the healing mechanism - Potential for pressure build-up within the network, causing system failure
Thermoplastics	- Simple to implement - Reversible - Multiple healing events possible	- Often requires external stimuli (like heat) to trigger healing - Healing may not be as effective at the nanoscale or for severe damage

## Data Availability

All data are contained within this article.
